# Advancing Musculoskeletal Care Using AI and Digital Health Applications: A Review of Commercial Solutions

**DOI:** 10.1177/15563316251341321

**Published:** 2025-05-30

**Authors:** Johannes Pawelczyk, Moritz Kraus, Sebastian Voigtlaender, Sebastian Siebenlist, Marco-Christopher Rupp

**Affiliations:** 1 Sports Orthopedics Department, Klinikum Rechts der Isar, Technical University of Munich, Munich, Germany; 2 Department of Traumatology, University Hospital Zurich, Zurich, Switzerland; 3 Virtual Diagnostics Unit, QuantCo, Inc., Cambridge, MA, USA

**Keywords:** artificial intelligence, digital health, machine learning, musculoskeletal care, commercial solutions

## Abstract

Artificial intelligence (AI) and digital health (DH) solutions are reshaping musculoskeletal (MSK) care across diagnostics, treatment planning, workflow optimization, and administrative burden reduction. AI-enabled triage systems enhance patient flow efficiency, while automated scheduling, symptom checkers, and AI-powered virtual assistants streamline pre-visit interactions. In MSK radiographic diagnostics, AI augments imaging interpretation, enabling automated fracture detection, opportunistic screening, and quantitative imaging, improving diagnostic accuracy and standardization. Preoperative planning solutions facilitate implant templating, surgical navigation, and patient-specific instrumentation, reducing variability and enhancing surgical precision. Concurrently, digital scribes and AI-driven documentation tools alleviate administrative overhead, mitigating clinician burnout and enabling refocused patient engagement. Predictive analytics optimize treatment pathways by leveraging multimodal patient data for risk stratification and personalized decision support. However, algorithmic bias, model generalizability, regulatory hurdles, and legal ambiguities present substantial implementation barriers, necessitating rigorous validation, adaptive governance, and seamless clinical integration. The U.S. and EU regulatory landscapes diverge in their approaches to AI oversight, with the former favoring expedited market access and the latter imposing stringent compliance mandates under the EU AI Act. AI’s integration into MSK care demands robust validation frameworks, standardized interoperability protocols, and dynamic regulatory pathways balancing safety and innovation. Emerging generalist foundation models, open-source large language models (LLMs), and specialized AI-driven medical applications herald a paradigm shift toward precision MSK care. These innovations will require prospective clinical validation to ensure patient benefit and mitigate risk. Addressing ethical considerations, ensuring equitable access, and fostering interdisciplinary collaboration remain paramount in translating AI’s potential into tangible improvements in MSK healthcare delivery.

## Introduction

Musculoskeletal (MSK) care is being transformed by advancements in artificial intelligence (AI), digital health (DH) technologies, and the increasing availability of healthcare data [43,44]. Over the past decade, considerable research has been directed to the application of AI in addressing the complex challenges associated with MSK care [12]. However, for this growing body of work to yield real-world benefits, translation into clinical practice is critical.

The need for such innovations is underscored by the rising prevalence of MSK disorders, driven largely by an aging population and the associated increase in chronic conditions, which continue to be a leading cause of disability worldwide [26]. This has led to a substantial administrative burden being placed on healthcare providers, leading to dissatisfaction and burnout [35]. Recent findings have demonstrated that for every hour of clinical face time with patients, nearly 2 additional hours are spent on electronic health record (EHR) completion and desk work, with another 1-2 hours of after-hours clerical tasks being reported [58]. DH solutions and AI technology have the potential to alleviate these issues and yield improved efficiency in clinical workflows, as well as enabling a new level of personalized care [22].

This review aims to provide an overview of current and emerging commercial AI and DH solutions relevant to MSK care, including software, devices, and platforms. The capabilities, limitations, and future potential of these technologies are examined, and practical applications, risks, and future opportunities are highlighted. In addition, the risks and challenges associated with the integration of AI and DH technology into clinical practice are addressed in an attempt to provide a balanced perspective on the opportunities for advancing MSK care through digital innovation. Although this review highlights many commercial products, we aim to provide a useful survey, realizing that many additional products exist, without endorsing the superiority of any platform.

## AI and Digital Health in Orthopedics

### Important Definitions

Understanding the relevant terminology is essential for discussing advancements in MSK care. AI is an umbrella term that can be defined as “intelligence demonstrated by machines” [1], including systems capable of learning, reasoning, and self-correction. Machine learning (ML), a core subset of AI, comprises “optimizing mathematical models to predict variables of interest from data” [66] without explicit programming. Finally, DH represents diverse digital tools, such as mobile apps, wearables, telemedicine, and EHRs, that improve healthcare delivery, but don’t necessarily rely on the advanced predictive capabilities associated with AI and ML.

### Regulatory Landscape and Standards

The regulatory landscape for AI in healthcare is characterized by distinct frameworks in the United States and the European Union (EU), each presenting distinct mechanisms for oversight and market entry, as well as unique opportunities and challenges.

In the United States, the Food and Drug Administration (FDA) has developed a comprehensive approach to the regulation of AI-enabled medical devices. Initially, AI was integrated into existing “software as medical device” approval frameworks, utilizing existing regulatory pathways, including “premarket approval,” “de novo,” and “510(k)” [3]. This approach facilitates market entry, particularly via the “510(k)” pathway, allowing devices to bypass more extensive evaluation if substantial equivalence to an approved predicate device is demonstrated. Furthermore, the FDA has issued specific guidance documents to address the unique challenges posed by AI technologies in the medical sector. In December 2024, the FDA finalized a guidance document on “Predetermined Change Protocol Plans”, allowing manufacturers to implement certain pre-authorized modifications to AI-enabled devices, without necessitating new submissions, thereby facilitating adaptive learning while maintaining safety and efficacy standards. Furthermore, the FDA emphasizes adherence to “Good Machine Learning Practice” principles, promoting the development of high-quality AI/ML-based medical devices.

In contrast, the European Union’s “EU AI Act” introduces a risk-based regulatory framework, mandating stringent compliance measures for high-risk AI systems, including third-party conformity assessments, post-market surveillance, and transparency obligations [5,52]. The legislation operates alongside the General Data Protection Regulation (GDPR), which governs AI-driven personal data processing, requiring stringent privacy safeguards, data minimization, and cross-border compliance mechanisms [5].

While the EU’s approach aims to enhance consumer protection and ethical oversight [52], its rigid and burdensome compliance framework risks both stifling innovation and eroding the EU’s competitive edge in medical AI development, where it currently holds a leadership position in patent ownership [5,34]. In contrast, the US’s system with its faster market access and lower compliance burden may foster a more dynamic AI innovation ecosystem, albeit at the cost of potentially weaker patient safety and ethical oversight mechanisms. As AI systems become increasingly integrated into healthcare, regulatory frameworks must balance risk mitigation with innovation enablement, ensuring that governance mechanisms remain agile, scalable, and attuned to emergent technological paradigms.

## Commercial AI and Digital Health Applications in Muskuloskeletal Care

**Fig. 1.**
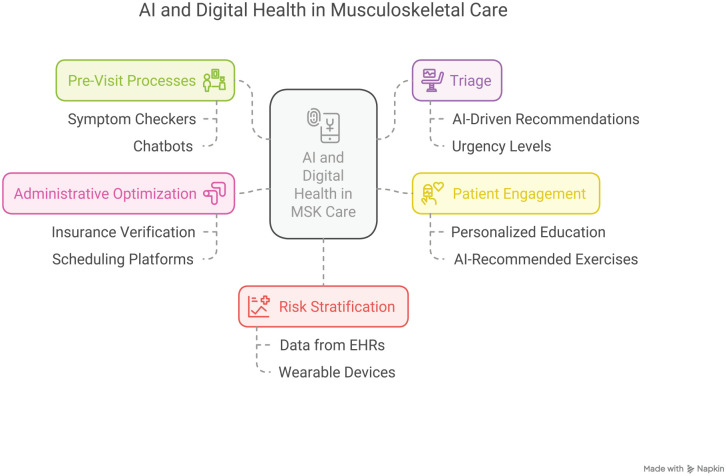
AI and Digital Health in MSK care. *AI*, artificial intelligence; *EHR*, electronic health record; *MSK*, musculoskeletal.

Advances in commercial AI and DH are transforming every stage of the MSK patient journey. Commercial stakeholders are developing innovative solutions to enhance care quality, boost efficiency, and elevate the patient experience. These technologies optimize pre-visit triage and patient intake, streamline clinical processes during appointments, and refine diagnostic imaging and surgical planning. Moreover, advanced predictive models support clinical decision-making while digital therapeutics are emerging to improve rehabilitation outcomes (Fig. 1). Collectively, these efforts underscore the potential of integrated digital solutions to optimize care delivery, reduce administrative burdens, and, ultimately, improve patient outcomes. While not all solutions are discussed in the main manuscript, additional examples are provided in Supplemental Table 1.

### Pre-visit and Triage

In the pre-visit stage, commercial solutions relying on AI and DH technologies aim to streamline triage processes and enhance patient engagement, setting a foundation for a more efficient care journey. AI-driven symptom checkers guide patients through structured symptom reporting, as well as generating differential diagnoses and triage recommendations (Supplemental Table 1). These tools may contribute to mitigating inappropriate self-triage—misjudged in ~60% of cases—potentially reducing unnecessary healthcare expenditures, which amount to an estimated $1 trillion USD [36]. Patients primarily use symptom checkers to understand symptom etiology, determine care necessity, and select appropriate healthcare levels, perceiving these tools to be highly usable and clinically beneficial [30,31]. Digital self-triage systems steer patient decision-making, with 15-30% of users adjusting care-seeking behavior in response to triage outputs [36]. Studies assessing diagnostic accuracy report heterogeneous findings. Certain commercially available solutions achieve performance comparable to human clinicians. One product demonstrated 70% diagnostic accuracy for inflammatory rheumatic diseases, outperforming physicians (54%, *p* = .002) and providing urgency advice on par with general practitioners [18,19]. However, performance has been shown to vary significantly across platforms [21], and an audit of 23 commercially available symptom checkers revealed inconsistencies in both triage and diagnostic accuracy, with a prevalent tendency toward risk-averse recommendations, frequently advising professional consultation where self-care would suffice [53].

Automated scheduling platforms such as Zocdoc (Zocdoc, Inc., New York, NY, US) and Doctolib (Doctolib GmbH, Berlin, Germany) optimize patient flow by allocating appointment slots based on urgency and forecasted visit lengths, offering convenient features to patients such as online booking and verified reviews. In addition, patients may be able to complete intake forms online and check their insurance coverage. For providers, these tools enable efficient scheduling, reducing no-shows, and attract new patients through increased visibility. Providers increasingly employ AI/ML features, for example, to enable conversational search using everyday language, facilitate insurance verification, or enable real-time provider-patient matching. Furthermore, some of these services integrate telehealth consultations, facilitating more accessible healthcare in the MSK sector in medically underserved areas [7,39].

### Patient Intake and Communication

During patient intake, seamless processes and effective communication ensure that patients are well-prepared for their appointments and clinical teams are equipped with the critical information needed for optimal care delivery. AI-enhanced intake platforms integrate with EHR systems to automate pre-registration, scheduling, payment processing, and insurance verification. One 4-hospital health system reported a 3.5-minute reduction in patient check-in time [4]. Recently, many start-ups and emerging companies have been focusing on developing more specialty-specific solutions. For MSK care, AllaiHealth (AllaiHealth, Inc., Atlanta, GA, US) employs AI-powered pre-visit interviews to optimize intake, pre-charting, and patient education, ensuring clinicians receive a structured, relevant patient profile before consultation. Similarly, OrthoCoPilot.AI, a dedicated MSK patient anamnesis tool, has demonstrated high levels of physician and patient satisfaction. Using this tool, 100% of doctors reported efficiency gains and benefits in information structuring, which positively influenced patient interaction in 83.3% of cases. Furthermore, the majority of patients (66.7%) felt that they and their doctors (73.3%) were better prepared for consultations [46].

Aaron.ai (Doctolib GmbH, Berlin, Germany), a digital practice assistant, is an example of an AI-powered chatbot being employed to improve patient communication. The chatbot automates patient communication, particularly phone-based interactions, through natural language interactions, handling appointment scheduling, prescription requests, and emergency triage. Operating 24/7, it aims to alleviate administrative burden by transcribing patient requests and integrating seamlessly with practice management systems while improving efficiency and reducing wait times.

### Doctor’s Appointment – Process Optimization and Clinical Documentation

AI and DH innovations aim to streamline documentation and administrative workflows surrounding clinical encounters, thus contributing to improved operational efficiency and reduced provider burden. Widespread burnout among healthcare professionals, largely driven by high workloads and administrative burdens, underscores the urgent need for efficiency-enhancing solutions [15,35]. AI-driven automation has the potential to minimize time-intensive and laborious tasks, allowing clinicians to prioritize care while deriving greater fulfillment from patient interactions [64].

Digital scribes (Supplemental Table 1) are designed to lessen the clinical documentation burden. These tools leverage speech recognition, natural language processing (NLP), and ML to transcribe nonstructured clinician-patient interactions into structured medical notes and allow for EHR integration. Substantial research interest has been directed toward this rapidly advancing field and several studies report promising clinical benefits [63]. To date however, there is no consensus regarding the advantages of these tools. For instance, 1 analysis of DAX Copilot (Microsoft Corporation, Redmond, WA, US) demonstrated significant reductions in task load and burnout [54]. However, another study at a large academic medical center found only marginal efficiency gains [29]. Other studies have also reported no statistically significant improvement in clinician efficiency, highlighting the uncertain real-world impact of these solutions [27] and suggesting results may differ between different clinical scenarios [65].

An example of a potentially superior AI clinical assistant, BotMD (5 Health, Inc., Anaheim, CA, US; cf. Supplemental Table 1), integrates hospital-specific content and workflows into a centralized platform, enabling instant access to critical information, such as protocols, drug formularies, or clinical guidelines via mobile or chat interfaces, eliminating the need to navigate complex intranet systems or contact hospital operators. Key functionalities include automated appointment scheduling, follow-up reminders, patient registration, and remote monitoring, reducing administrative burdens. In addition, it offers clinical decision support, including medical calculators and AI-assisted drug dosing, and provides multilingual patient engagement solutions. The platform integrates seamlessly with hospital systems, ensuring real-time access to important clinical information.

### Radiology and Imaging

Diagnostic imaging is a fundamental pillar of MSK care, providing critical insights that guide diagnosis and treatment. At the same time, notable operational challenges persist, with existing radiological workflows often being time-consuming, subjective, and non-standardized, while clinicians are faced with increasing workload complexity, documentation requirements, and burnout [8,15]. The integration of AI into this domain has gained considerable traction, with 79% of FDA-authorized AI devices targeting radiological applications [60]; further, AI has shown promise in alleviating the abovementioned issues [25,51,56].

AI-driven **computer-aided triage and diagnostic systems** represent a primary application domain. FDA-cleared solutions have demonstrated clinical utility across varied anatomical regions by automatically flagging suspected pathologies and prioritizing urgent cases. These systems typically operate through automatic background analysis of imaging studies, flagging critical findings such as vertebral compression fractures, cervical spine fractures, and general MSK trauma, thus claiming significant workflow improvements, as well as reductions in turnaround time and false negatives (Supplemental Table 1). However, independent clinical studies often demonstrate only moderate diagnostic performance [59,67].

AI-powered **opportunistic screening** enables concurrent evaluation for secondary conditions, particularly in high-risk populations. FDA-approved solutions, such as Rho (16 Bit, Inc., Toronto, ON, Canada), facilitate the identification of potential osteoporosis in patients >50 years through analysis of routine radiographs. FLAMINGO (IB Lab GmbH, Vienna, Austria) enables standardized detection of vertebral fractures as incidental findings on computed tomography (CT) imaging. Notably, Rho was found to have very good correlations (AUCs of 0.82-0.89) for the detection of low bone mineral density, with consistent performance across demographics and anatomical sites [9].

AI-based **quantitative imaging** applications aim to address subjectivity and inconsistency in radiological assessments through automated measurement and segmentation. While there already is substantial academic evidence [14,69], FDA- and Conformité Européene (CE)-marked solutions provide additional safety in standardized evaluations of specific pathologies. KOALA’s (IB Lab GmbH, Vienna, Austria) automated assessment of knee osteoarthritis using Osteoarthritis Research Society International criteria and Kellgren-Lawrence grading, for example, improved physician’s agreement rate with a gold standard [37]. Similar solutions exist, targeting spinal morphology (SQUIRREL, IB Lab GmbH, Vienna, Austria), hip morphology (HIPPO, IB Lab GmbH, Vienna, Austria), and limb alignment (LAMA, IB Lab GmbH, Vienna Austria). These tools demonstrate particular utility in conditions requiring tedious or precise geometric measurements. As such, SQUIRREL exhibited excellent results in automated Cobb angle measurement, with a mean difference from the reference standard of only 0.16°. In this instance, SQUIRREL outperformed a panel of radiologists [13]. Correspondingly, AI-based solutions have shown significant potential for time savings compared to manual annotation, with HIPPO reportedly reducing measurement time when extracting 6 hip measurements from AP radiographs from 6 minutes to just 41 seconds [6]. Promising mobile applications include Orthelligent Vision (OPED GmbH, Valley/Oberlaindern, Germany) for precise, marker-less gait analysis, and Momentum Spine (Momentum Health, Montreal, QC, Canada) for scoliosis assessment; these provide detailed quantitative measurements without requiring specialized equipment and potentially enable use cases such as remote disease monitoring.

Similarly, **preoperative planning** is an important component of presurgical workflows, associated with increased surgeon confidence [45], decreased cost [55], and even improved outcomes [32]. However, preoperative planning can be associated with significant additional time expenditure and tedious manual measurements [32]. AI-enabled solutions have demonstrated the ability to significantly enhance accuracy and reduce templating times [33]. Tools such as SMART Bun-Yo-Matic (Paragon 28, Inc., Englewood, CO, US) and HealthJOINT (Zebra Medical Vision Ltd., Shefayim, Israel) provide 3-dimensional (3D) reconstructions or reports from 2D radiographs, potentially reducing the reliance on 3D imaging. Standalone solutions such as Preview Shoulder (Genesis Software Innovations, Grand Rapids, MI, US) enable preoperative planning based on CT imaging in the context of shoulder arthroplasty, allowing the surgeon to digitally place the implant in the patient’s anatomy and generating a detailed report. Additive manufacturing applications, such as Axial3D (Axial3D, Belfast, United Kingdom) and Acorn 3D Software (Mighty Oak Medical, Englewood, CO, US), further extend AI’s role by enabling 3D-printed anatomical replicas for preoperative rehearsal and intraoperative reference, as well as for patient education. They may also allow for the construction of patient-specific surgical instrumentation to provide intraoperative support based on preoperative planning, respecting individual patient anatomy (e.g., United Orthopedic Knee Patient Specific Instrumentation, Precision AI Surgical Planning System, and SMART Bun-Yo-Matic). Furthermore, existing integrated solutions combine planning capabilities with intraoperative navigation and postoperative verification. Increasingly, implant manufacturers have recognized the importance of this technology and have expanded their portfolio to offer integrated preoperative planning software solutions such as Arthrex VIP (Arthrex, Inc., Naples, FL, US) or Stryker Blueprint (Stryker Corporation, Kalamazoo, MI, US) to enable preoperative planning, simulation of postoperative range of motion, or patient-specific instrumentation.

AI-enabled **surgical navigation and intraoperative support** systems extend AI’s utility to the operating room. An analysis of an existing integrated planning and intraoperative navigation solution found excellent performance in the context of total hip arthroplasty, with high precision in anteversion, inclination, and cup position depth [38]. Other commercially available solutions aim to assist surgeons during hip arthroscopy for femoroacetabular impingement by providing quantitative intraoperative measurements (e.g., alpha angle). Concurrently, the emergence of augmented reality systems (cf. Supplemental Table 1) represents an evolution in surgical guidance, providing hands-free navigation during arthroplasty procedures through marker tracking and spatial registration. By integrating high-resolution preoperative imaging into the surgical workflow, these solutions not only enhance intraoperative accuracy and reliability but also naturally pave the way for robotic-assisted products like Stryker Corporation’s MAKO, DePuy Synthes’ VELYS (DePuy Synthes, Raynham, MA, US), ROSA (Zimmer Biomet Holdings, Warsaw, IN, US), or CORI (Smith & Nephew PLC, London, United Kingdom) systems. Systematic review data have shown that such imaging significantly reduces mechanical axis alignment outliers. In 1 meta-analysis, 14 out of 16 radiological studies demonstrated superior implant positioning compared to conventional methods [23], setting the stage for next-generation robotics systems that offer more predictable and patient-specific surgical outcomes.

Beyond image analysis, various **workflow optimization** tools have emerged, aimed at enhancing operational efficiency. AI-powered radiology report generation represents a primary use case. Such systems have significant potential to reduce report generation times, improve report quality, reduce errors, and enhance readability [41,61]. Rad AI Suite (Rad AI, San Francisco, CA, US) leverages NLP to generate structured radiology reports, claiming a reduction in dictation time by up to 50%. RadioReport Automatic AI (Neo Q Quality in Imaging GmbH, Berlin, Germany) achieves 50% faster reporting via guided virtual interviews and mandatory plausibility checks, according to the manufacturer. Finally, tools such as myExam Companion (Siemens Healthineers AG, Erlangen, Germany) use AI to standardize patient positioning and scan planning in magnetic resonance imaging/CT, reducing operator-dependent errors and scan times, helping users achieve reproducible results and guiding operators through diagnostic procedures.

On a cautionary note, the AI-physician relationship remains complex and improper AI implementation may exacerbate issues instead of alleviating them. A recent survey of 6726 radiologists reported an *increased* risk of burnout with frequent AI use, particularly among radiologists reporting high workloads and low AI acceptance [25].

### Predictive Models

In selecting the optimal treatment option for each patient, ML models are increasingly utilized to analyze complex healthcare datasets, inform clinical decision-making, optimize resource allocation, and predict outcomes [43,48]. Predictive analytics support data-driven care pathways, aligning physician decision-making with cost-effectiveness and patient benefit. These models also enable risk stratification by estimating complication probabilities, reinjury likelihood, and implant durability, thereby aiding preoperative planning and post-treatment monitoring. In the future, commercial AI solutions may further enhance MSK care quality through AI-driven risk stratification while also refining insurers’ systems for premium adjustments, treatment approval decisions, and potentially, value-based contracts that incentivize long-term positive outcomes.

Technology-enabled health services such as Optum (Optum, Inc., Eden Prairie, MN, US), integrate predictive modeling to enhance care delivery and reduce costs. For example, Optum’s “Next Best Action” framework synthesizes clinical, behavioral, and demographic data to recommend personalized interventions, dynamically optimizing treatment selection and patient engagement strategies based on projected outcomes. As such, for patients with similar symptoms but different health histories, predictive models may prioritize certain treatments based on projected clinical value and individual patient engagement likelihood. Furthermore, Epic’s EHR-integrated predictive analytics evaluate clinical and operational data to anticipate disease progression, complications, and care needs. These models support preventative strategies, optimize patient flow, predict staffing requirements, and streamline administrative processes, including billing automation. Population health management tools enable proactive outreach for at-risk groups, while analytics platforms such as “Slicer Dicer” and “Clarity” facilitate large-scale data-driven insights for clinical research and operational efficiency.

### Therapeutic Devices and Rehabilitation

In the realm of physical therapy and rehabilitation following any surgical or nonsurgical orthopedic treatment, DH solutions offer targeted interventions that address rising demands for physiotherapy services amid workforce challenges. MSK solutions like Companion Patella and Companion Shoulder (medi GmbH & Co. KG, Bayreuth, Germany) provide targeted rehab tools, while ViViRA (ViViRA Health Lab GmbH, Berlin, Germany), a prescription-based app, has demonstrated a significant reduction in pain intensity. In a randomized trial, patients with nonspecific and degenerative back pain using ViViRA experienced a clinically relevant mean pain reduction of 53.1% over 12 weeks, compared to 14.6% in the standard physiotherapy group [68]. Other digital platforms offer personalized rehabilitation programs with progress tracking, primarily designed to improve adherence to home-based exercise programs [49]. Positive real-world effects of a digital prescription home exercise program for degenerative MSK conditions have been demonstrated [62]. In the United States, services such as Thrive (Sword Health, Inc., Draper, UT, US) or Hinge Health (Hinge Health, Inc., San Francisco, CA, US) offer AI-powered physical therapy platforms, providing feedback and data collection during therapy sessions and allowing providers to remotely monitor progress and adjust treatment plans, claiming to provide “world-class care” from the comfort of patients’ homes.

Still, low prescription rates and inherent financial risks remain, making payors skeptical about DH applications [20]. To improve access and adherence, further education, simplified processes, and streamlining patients’ ability to seek treatment with digital therapeutics are needed [16]. Europe is adopting centralized pathways to regulate mHealth applications, with Germany leading in introducing a legal framework for digital app certification, including DH applications in the statutory health insurance system in late 2019, making them eligible for reimbursement by public insurance [20].

## Benefits and Opportunities

Physician burnout is driven by many factors, including administrative and documentation burden [63]. Ambient AI scribes and AI-enabled radiology report generation solutions have shown promise in reducing these workloads while enhancing report quality and readability [29,41,54,61,63]. Similarly, advanced scheduling solutions streamline workflows by automating manual tasks and improving appointment booking, allowing physicians to engage more meaningfully with patients. These innovations may improve doctors’ job satisfaction [10], boost operational efficiency, and enhance the overall patient experience through accelerated diagnostics and reduced wait times. Taken together, these benefits might allow physicians and patients to engage more meaningfully and reclaim time and energy to strengthen their patient-physician relationship [63]. McKinsey & Company estimates that up to 45% of administrative tasks could be automated, enabling annual cost savings of approximately $150 billion USD in the United States alone [2].

Beyond these workflow improvements, AI-augmented imaging interpretation, opportunistic screening, risk stratification, and AI-powered preoperative planning and surgical support are paving the way for individualized interventions that help to improve clinical outcomes. By integrating multimodal patient data, AI facilitates a shift from traditional “mean value thinking” [22] toward individualized care, promising more precise, patient-specific interventions that enhance both short- and long-term outcomes. Furthermore, digital tools such as telehealth solutions and AI symptom checkers broaden access to high-quality care, enabling more equitable healthcare delivery and reducing geographical disparities.

Looking ahead, advances in open-source large language models such as Meta’s Llama series or DeepSeek’s recently released open-source R1 reasoning model, which outperformed state-of-the-art proprietary models such as OpenAI’s o1 on several key benchmarks, and the increasing commoditization of high-performance compute infrastructure, exemplified by Nvidia’s DIGITS project, promise unprecedented on-premises research and deployment capabilities, circumventing privacy concerns and simplifying regulatory compliance. Recent breakthroughs in the generalist medical foundation model domain, such as Google’s MedPaLM 2 [57], offer an exciting glimpse into a future where healthcare is augmented by AI models with generalist medical capabilities approaching those of human physicians across several dimensions such as medical question answering.

## Risks, Limitations, and Ethical Considerations

AI and DH applications in MSK care offer significant promise but also introduce notable risks and ethical challenges. Algorithmic biases from skewed training data can worsen healthcare disparities and trigger diagnostic errors, underscoring the need for rigorous validation and continuous monitoring [11,42]. Overreliance on AI may degrade clinical judgment [24], while surveys among orthopedic surgeons highlight concerns over diminishing skills in the next generation due to excessive dependence on technology [47]. Technical limitations—such as performance decay under distribution shifts and limited generalizability—combined with insufficient external validation, further compromise reliability [17,70]. In addition, the management of extensive patient data raises privacy and security concerns, increasing vulnerability to cybersecurity threats and complicating compliance with frameworks like GDPR and HIPAA. Legal ambiguities regarding liability for AI-driven decisions, along with high costs, integration challenges, and interoperability deficits, hinder clinical adoption, while provider resistance and patient mistrust—fueled by fears of depersonalized care—exacerbate these challenges. Ethically, AI must augment rather than replace human expertise, ensuring transparency in decision-making and preserving patient-physician trust. Addressing these issues will require interdisciplinary collaboration, robust validation frameworks, and adaptive regulatory pathways that balance innovation with safety and equity.

## Strategies for Successful Integration

**Fig. 2.**
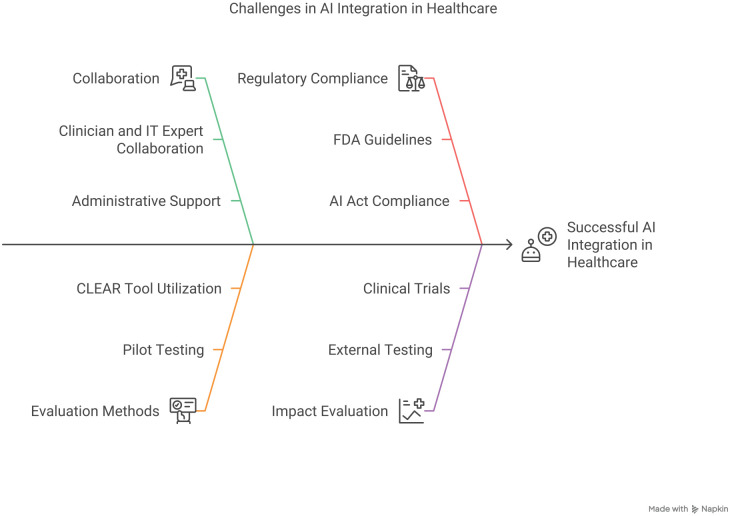
Challenges in AI Integration in Healthcare. *AI*, artificial intelligence; *FDA*, Food and Drug Administration; *IT*, information technology.

Successful integration of AI and DH in MSK care demands a comprehensive, interdisciplinary approach (Fig. 2). Rigorous pilot testing and validation on diverse, independent cohorts are essential to ensure robust real-world performance and mitigate issues such as performance decay [17]. Transparent reporting—guided by standardized metrics such as the CLEAR tool and CONSORT-AI [28,50]—can facilitate objective assessment and wider clinical adoption. Continuous education and structured communication among clinicians, administrators, and other stakeholders are critical for aligning expectations and managing operational challenges. Moreover, adherence to evolving regulatory standards from bodies such as the FDA and European Medicines Agency (EMA) [40], combined with strategies to overcome interoperability and scalability barriers, is vital. A collaborative framework that engages healthcare providers, engineers, ethicists, policymakers, and legal experts will ensure that AI innovations are integrated safely and effectively, ultimately translating into improved clinical outcomes and more equitable access to MSK care.

## Conclusion

AI and DH technologies are transforming MSK care by enhancing diagnostic accuracy, optimizing workflows, and alleviating administrative burdens. AI-driven triage, imaging, predictive modeling, and surgical planning may streamline clinical decision-making, while digital scribes and automation tools can be used in efforts to mitigate clinician burnout. However, challenges persist, including algorithmic bias, regulatory uncertainty, data privacy concerns, and integration hurdles. Ensuring rigorous validation, regulatory adaptability, and seamless clinical adoption is critical. Future efforts must prioritize interoperability, prospective evaluation, and ethical AI deployment to maximize patient benefit while maintaining clinician oversight. As AI capabilities evolve, a balanced approach fostering innovation without compromising safety and equity will be essential in realizing AI’s full potential in MSK healthcare.

## Supplemental Material

sj-docx-1-hss-10.1177_15563316251341321 – Supplemental material for Advancing Musculoskeletal Care Using AI and Digital Health Applications: A Review of Commercial Solutions Supplemental material, sj-docx-1-hss-10.1177_15563316251341321 for Advancing Musculoskeletal Care Using AI and Digital Health Applications: A Review of Commercial Solutions by Johannes Pawelczyk, Moritz Kraus, Sebastian Voigtlaender, Sebastian Siebenlist and Marco-Christopher Rupp in HSS Journal®

sj-docx-2-hss-10.1177_15563316251341321 – Supplemental material for Advancing Musculoskeletal Care Using AI and Digital Health Applications: A Review of Commercial Solutions Supplemental material, sj-docx-2-hss-10.1177_15563316251341321 for Advancing Musculoskeletal Care Using AI and Digital Health Applications: A Review of Commercial Solutions by Johannes Pawelczyk, Moritz Kraus, Sebastian Voigtlaender, Sebastian Siebenlist and Marco-Christopher Rupp in HSS Journal®

sj-docx-3-hss-10.1177_15563316251341321 – Supplemental material for Advancing Musculoskeletal Care Using AI and Digital Health Applications: A Review of Commercial Solutions Supplemental material, sj-docx-3-hss-10.1177_15563316251341321 for Advancing Musculoskeletal Care Using AI and Digital Health Applications: A Review of Commercial Solutions by Johannes Pawelczyk, Moritz Kraus, Sebastian Voigtlaender, Sebastian Siebenlist and Marco-Christopher Rupp in HSS Journal®

sj-docx-4-hss-10.1177_15563316251341321 – Supplemental material for Advancing Musculoskeletal Care Using AI and Digital Health Applications: A Review of Commercial Solutions Supplemental material, sj-docx-4-hss-10.1177_15563316251341321 for Advancing Musculoskeletal Care Using AI and Digital Health Applications: A Review of Commercial Solutions by Johannes Pawelczyk, Moritz Kraus, Sebastian Voigtlaender, Sebastian Siebenlist and Marco-Christopher Rupp in HSS Journal®

sj-docx-5-hss-10.1177_15563316251341321 – Supplemental material for Advancing Musculoskeletal Care Using AI and Digital Health Applications: A Review of Commercial Solutions Supplemental material, sj-docx-5-hss-10.1177_15563316251341321 for Advancing Musculoskeletal Care Using AI and Digital Health Applications: A Review of Commercial Solutions by Johannes Pawelczyk, Moritz Kraus, Sebastian Voigtlaender, Sebastian Siebenlist and Marco-Christopher Rupp in HSS Journal®

sj-pdf-6-hss-10.1177_15563316251341321 – Supplemental material for Advancing Musculoskeletal Care Using AI and Digital Health Applications: A Review of Commercial Solutions Supplemental material, sj-pdf-6-hss-10.1177_15563316251341321 for Advancing Musculoskeletal Care Using AI and Digital Health Applications: A Review of Commercial Solutions by Johannes Pawelczyk, Moritz Kraus, Sebastian Voigtlaender, Sebastian Siebenlist and Marco-Christopher Rupp in HSS Journal®
