# Supplementary material for: Advancing Musculoskeletal Care Using AI and Digital Health Applications: A Review of Commercial Solutions
Source: HSS J. 2025 May 30;21(3):331–41. doi: 10.1177/15563316251341321 (PMC12126469; doi:10.1177/15563316251341321)
Supplement: sj-docx-1-hss-10.1177_15563316251341321 – Supplemental material for Advancing Musculoskeletal Care Using AI and Digital Health Applications: A Review of Commercial Solutions [file sj-docx-1-hss-10.1177_15563316251341321.docx]

**Supplemental Table 1.** Non-comprehensive overview of commercial AI solutions in orthopedics and beyond. Solutions are classified as AI-based (AI), classical digital health solution (DH), or a combination of both.

| **Product** | **Company** | **AI / DH** | **Category** | **Subcategory** | **Description** |
| --- | --- | --- | --- | --- | --- |
| *Pre-visit and triage* | | | | | |
| [Ada DX / Assess](https://ada.com/) | Ada Health GmbH | AI | Symptom checker | n/a | Browser-based online tool/smartphone app, available direct to consumer; guides patients through symptom inputs to determine a “triage” recommendation, suggesting the level of urgency, directs user to appropriate care options; also lists “possible causes”, i.e., *potential* diagnoses; CE-certified Class IIa medical device under EU’s MDR |
| [Buoy](https://www.buoyhealth.com/) | Buoy Health Inc. | AI | Symptom checker | n/a | AI-driven browser-based tool with chat-like interface; guides patients through structured symptom inputs; recommends care pathways; positioned as a consumer health information tool, rather than a regulated medical device |
| [Isabel](https://symptomchecker.isabelhealthcare.com/) | Isabel Healthcare Ltd. | AI | Symptom checker | n/a | AI-powered symptom checker and clinical decision support tool; utilizes AI to provide diagnostic assistance and triage recommendations, covering a wide array of diseases/pathologies |
| [Symptomate](https://symptomate.com/) | Infermedica | AI | Symptom checker | n/a | AI-powered self-service symptom checker and DH assistant (web-based/smartphone app); guides user through structured interview and also offers a chatbot-based interface; Class I medical device certification under EU’s MDR, FDA-regulated medical device (general wellness product) in the U.S.; HIPAA/GDPR compliant |
| [Avey](https://avey.ai/) | Rimads QSTP LLC | AI | Symptom checker | n/a | AI-powered app-based symptom checker and healthcare platform; uses AI technology to provide accurate self-diagnosis and health assessments; also offers appointment booking and communities to connect with other users |
| [Zocdoc](https://www.zocdoc.com/) | Zocdoc Inc. | DH | Appointment booking | n/a | Appointment booking (in-person or telemedicine) and scheduling system, with filters for insurance coverage; automates timeslot matching by location, specialty, and insurance; provides reviews/ratings; free for patients, doctors pay to advertise their appointment slots; check-in feature added in 2012, allowing patients to fill out medical forms online prior to visit |
| [Doctolib](https://www.doctolib.com/) | Doctolib SAS | DH | Appointment booking | n/a | Web-based application/smartphone app; widely used for appointment booking/scheduling and practice management in Germany and France; no ratings/reviews; provides access to telehealth video consultations |
| [Sharecare+](https://www.sharecare.com/solutions/sharecare-plus-landing-page) | Sharecare Inc. | AI | Screening / risk stratification | n/a | Digital health platform offering whole person risk analytics and advanced user segmentation to identify opportunities for deeper engagement and risk reduction aimed at employee monitoring; offers health risk assessments and biometric screenings; recently acquired by Altaris for $518 million USD (announced 10/2024) |
| [OptumIQ](https://www.optum.com/en/) | Optum Inc. | AI | Screening / risk stratification | n/a | Centralized data and AI platform, leveraging data analytics, natural language processing, and ML/DL models to improve patient outcomes and decrease care costs; offers risk stratification tools for payors and providers, provides insurers and healthcare providers with predictive models to guide coverage decisions; Optum Inc. is part of UnitedHealth Group |
| [DocRobin](https://docrobin.net/) | Medical Intelligence Lab GmbH | AI | Patient education | n/a | Animated AI physician avatar or chatbot guides patients through information intake; AI can analyze radiographs uploaded by the patient; provides evidence-based second opinion regarding decision for/against TKA from a qualified physician within 48 hours; recently announced collaboration with ‘Techniker Krankenkasse’ (large German insurer) |
| *Doctor’s appointment – process optimization, telehealth, and clinical documentation* | | | | | |
| [Phreesia](https://www.phreesia.com/) | Phreesia Inc. | DH | Process optimization | Patient intake management | Automates patient intake and other administrative tasks; verifies insurance eligibility; in 2021, acquired schedule management solution QueueDr (now Phreesia Appointment Accelerator), as well as Insignia Health; in 2023, acquired Sulphur Springs |
| [Bot MD](https://www.botmd.io/) | 5 Health Holdings Inc. | AI | Process optimization | Information management | Core technology is proprietary AI chat engine that can integrate with a wide range of hospital information systems to provide instant answers to doctor/patient queries; Bot MD Care is an AI patient monitoring platform that helps clinical care teams monitor and triage patient populations and enables video teleconsultations via popular chat platforms like WhatsApp, Viber, or Messenger; offers appointment scheduling capabilities, but also aims to automate many other manual workflows, such as patient engagement/education, remote monitoring, or clinical information search; Bot MD Hospital allows providers to instantly search their hospital’s information within a single chat interface; other use-cases include PROM collection, pre/post visit data collection, automated follow-up, handling patient inquiries, and chronic disease management |
| [AllaiHealth](https://allaihealth.com/) | AllaiHealth Inc. | AI | Process optimization | Patient intake management | Patient medical history platform; intended to optimize healthcare workflows by eliminating pre-charting and providing smart pre-visit screening, automated documentation, and patient education; ensures clinicians receive a structured, relevant patient profile before consultations |
| OrthoCoPilot | OrthoCoPilot.AI | AI | Process optimization | Patient intake management | Dedicated digital anamnesis tool for MSK care; guides patients through a proprietary, dynamic questionnaire based on the affected anatomic region, gathering all relevant information prior to patient-physician interaction; provides a detailed overview of important anamnestic findings to clinicians |
| [Aaron.ai](https://www.aaron.ai/) | Aaron GmbH | AI | Process optimization | Digital telephone assistant | Digital telephone assistant, ensures clinic/practice is always reachable via phone, reduces workload for human staff; used in Germany, Austria, and Switzerland since 2018; AI agent answers the phone when patients call the clinic/practice; inquiries are categorized and saved into a worklist/dashboard to be reviewed by a human; acquired by Doctolib in 2024 |
| [HealthTap](https://www.healthtap.com/) | HealthTap Inc. | DH | Telehealth | n/a | Delivers telehealth online primary care via web interface/smartphone app; offers option to immediately connect to, or schedule an appointment with, a physician for a consultation via video conference, phone call, or chat; supplied by a network of ~140,000 licensed doctors in good standing; offers “peer review” amongst physicians for quality assurance |
| [Suki Assistant](https://www.suki.ai/) | Suki AI Inc. | AI | Virtual scribe | n/a | AI-powered voice assistant for healthcare professionals, capturing clinical notes ambiently, by listening to patient-clinician interactions I real-time; supports EHR integration and voice commands; recommends codes and offers question answering, available for iOS, Android, web, and desktop; SOC2 Type 2 certification and HIPPA compliant |
| [DeepScribe Notes / DeepScribe Assist](https://www.deepscribe.ai/) | DeepScribe Inc. | AI | Virtual scribe | n/a | Automates note creation from exam room conversations using NLP; ambient AI documentation (DeepScribe Notes); offers real-time guidance/recommendations to physicians at the point of care (DeepScribe Assist); offers custom AI models for different specialties, incl. orthopedics; offers automatic coding for optimized billing |
| [DAX Copilot](https://www.nuance.com/healthcare/dragon-ai-clinical-solutions/dax-copilot.html?srsltid=AfmBOopqoyb06Avup9B6kyFII_iBAPUKjCqNeAjRQ2GzeNwfxcnIjChg) | Nuance Communications Inc. | AI | Virtual scribe | n/a | Ambient voice technology for real-time transcription; additional tools to streamline workflows and maximize administrative burden relief; uses Microsoft Cloud for Healthcare; EHR integration |
| [CodeCare](https://codecare.ai/) | CodeCare.ai | AI | Virtual scribe | n/a | AI-powered platform to streamline clinical documentation; works entirely in the browser; provides real-time documentation and specialty specific templates/workflows; offers EHR compatibility; early stage (currently beta access) |
| [Nabla Copilot](https://www.nabla.com/) | Nabla Technologies | AI | Virtual scribe | n/a | Ambient AI assistant; intended to alleviate documentation burden; features include EHR integration, medical coding, pre-charting, clinical notes, clinical decision prompting, and patient summaries; supports 55+ specialties and customizable templates, as well as multiple languages; SOC2 Type 2 certification and HIPAA compliant |
| *Diagnosis and imaging* | | | | | |
| [Aidoc](https://www.aidoc.com/) | Aidoc Medical Ltd. | AI | Radiology | Diagnostics / Triage | Computer-aided triage and notification system; FDA/CE mark approval for 20+ algorithms, incl. cervical spine fractures, rib fractures, and vertebral compression fractures; ‘always-on AI’ runs constantly in the background and automatically analyzes medical imaging data, identifying, flagging, and prioritizing urgent findings |
| [CINA-VCF](https://avicenna.ai/) | Avicenna.AI | AI | Radiology | Diagnostics / Triage | Computer-aided triage and notification system for the analysis of CT scans of patients >50 years which include the chest and/or abdomen; FDA approved; flags and communicates suspected positive cases of vertebral compression fractures; intended to assist with triage / prioritization; according to company homepage, solution leads to reduced interpretation time, optimized patient management, and enhanced radiology workflows |
| [CINA-CSpine](https://avicenna.ai/) | Avicenna.AI | AI | Radiology | Diagnostics / Triage | FDA-approved computer-aided triage and notification system for the analysis of CT scans of the cervical spine; automatically flags suspected fractures and alerts radiologists |
| [HealthVCF](https://www.accessdata.fda.gov/cdrh_docs/pdf19/K192901.pdf) | Zebra Medical Vision Ltd. | AI | Radiology | Diagnostics / Triage | Passive notification and prioritization software; FDA approved; flags patients with suspected vertebral compression fractures on chest / abdominal CTs; does not send a proactive alert directly to the user, does not provide diagnostic information beyond triage / prioritization |
| [AZtrauma](https://www.azmed.co/) | AZmed SAS | AI | Radiology | Diagnostics / Triage | FDA- and CE mark-approved computer-assisted diagnosis software to assist physicians in detection of fractures on MSK radiographs, improving workflow efficiency, diagnostic precision, and workload burden; company claims 83% turnaround time reduction, 67% false negatives reduction, and 99.7% negative predictive value; according to the company homepage, their software has been deployed in over 55 countries / 2,500 healthcare centers and has analyzed >15 million x-rays over the last year |
| [AZchest](https://www.azmed.co/) | AZmed SAS | AI | Radiology | Diagnostics / Triage | Clinically validated, CE mark-approved computer-assisted diagnosis software; automatically categorizes, detects, and reports key cardiac and pulmonary abnormalities, as well as rib fractures |
| [qMSK](https://www.qure.ai/product/qmsk) | Qure.ai Technologies Private Ltd. | AI | Radiology | Diagnostics / Triage | FDA and CE mark approved AI assistant for trauma MSK x-rays; automatically detects fractures, supporting 15 anatomies with multiple views; according to company homepage, processing time is under 20 seconds, with a >90% sensitivity to detect signs of fracture; offers ability to track progress in fracture healing, as well as worklist prioritization and auto filters |
| [BoneView](https://www.gleamer.ai/solutions/boneview) | Gleamer | AI | Radiology | Diagnostics / Triage | FDA- and CE mark-approved software intended to analyze radiographs using ML to identify and highlight fractures; intended as a concurrent reading aid; according to company website, >30 million examinations are analyzed annually in 2000+ public and private institutions, with >30 clinical studies |
| [RBfracture](https://www.imagebiopsy.com/product/rbfracture?utm_lang=en) | IB Lab GmbH | AI | Radiology | Diagnostics / Triage | Computer-aided diagnosis software to optimize fracture diagnosis in emergency departments, improving operational efficiency, and reducing turnaround times; automatic fracture detection on radiographs, covering most common MSK areas; highlights relevant findings by applying the latest international medical standards; findings are summarized in a visual report |
| [Rho](https://www.16bit.ai/rho) | 16 Bit Inc. | AI | Radiology | Opportunistic screening | FDA-approved software to be used opportunistically with standard frontal radiographs of the lumbar/thoracic spine, chest, pelvis, knee, or hand/wrist in patients > 50 years; provides notifications to aid in identifying patients with possible low bone mineral density / osteoporosis to prompt clinical bone health assessment |
| [FLAMINGO](https://www.imagebiopsy.com/product/flamingo?utm_lang=en) | IB Lab GmbH | AI | Radiology | Opportunistic screening | Computer-aided diagnostic software for opportunistic screening; provides accurate detection and labeling of vertebral fractures on CT scans; enables standardized detection of vertebral fractures as secondary radiological findings; provides vertebral labeling of thoracic and lumbar spine, as well as a graphical summary of all findings; limited to adults > 50 years |
| [KOALA](https://www.imagebiopsy.com/product/koala-fda?utm_lang=en) | IB Lab GmbH | AI | Radiology | Quantitative imaging | FDA- and CE mark-approved knee osteoarthritis labeling assistant; fully automated image processing software to aid in the measurement of the presence / absence of sclerosis, joint space narrowing, and osteophytes based on OARSI criteria, as well as the presence / absence of radiographic knee OA based on KL grading; provides a visual report summarizing the findings and facilitates monitoring of disease progression over time |
| [DeepXray Genu](https://www.aimfolds.com/deepxray-genu/) | Alpha Intelligence Manifolds Inc. | AI | Radiology | Quantitative imaging | FDA-cleared fully automated image processing software intended to aid in the assessment of presence / absence of sclerosis, joint space narrowing, and osteophytes based on OARSI criteria, as well as presence / absence of radiographic knee OA based on KL grading |
| [DeepXray Coxa](https://www.aimfolds.com/deepxray-coxa/) | Alpha Intelligence Manifolds Inc. | AI | Radiology | Quantitative imaging | AI-assisted diagnosis system; enables automated diagnosis and monitoring of osteoporosis based on x-rays |
| [FROG](https://www.imagebiopsy.com/product/ib-lab-frog?utm_lang=en) | IB Lab GmbH | AI | Radiology | Quantitative imaging | Certified medical software; provides automatic expert level foot x-ray measurements, enhancing reliability and reducing manual workload; aids in diagnosis and treatment planning; automatically extracts several important measurements, incl. hallux valgus angle, intermetatarsal angle, distal metatarsal articular angle, etc. |
| [SQUIRREL](https://www.imagebiopsy.com/product/squirrel?utm_lang=en) | IB Lab GmbH | AI | Radiology | Quantitative imaging | Enhances reliability and precision of Cobb angle measurements; fully automated software to support physicians in the assessment of spinal morphology and scoliosis evaluation using frontal radiographs of the spine (particularly for the purpose of scoliosis assessment and disease progression); automatically measures relevant scoliosis parameters, like the Cobb angle and coronal balance |
| [HIPPO](https://www.imagebiopsy.com/product/hippo-ce) | IB Lab GmbH | AI | Radiology | Quantitative imaging | Hip positioning assistant; enhances efficiency when reading pelvis radiographs; supports objective and standardized measurement of most important hip angles, incl. the CCD angle, LCE angle, pelvic obliquity, Tönnis angle, sharp angle, and femoral head coverage / extrusion index; fully automated |
| [LAMA](https://www.imagebiopsy.com/product/lama-ce) | IB Lab GmbH | AI | Radiology | Quantitative imaging | Leg angle measurement assistant; enables fully automated measurements to quantify limb-length discrepancy and quantitative knee alignment parameters on uni- and bilateral AP full leg radiographs |
| [MEDO ARIA](https://www.accessdata.fda.gov/cdrh_docs/pdf20/K200356.pdf) | Medo.ai | AI | Radiology | Quantitative imaging | Designed to view and quantify ultrasound image data using ML to aid in diagnosis of developmental dysplasia of the hip; intended to be used on neonates and infants aged 0-12 months |
| [PANDA](https://www.imagebiopsy.com/product/panda-ce) | IB Lab GmbH | AI | Radiology | Quantitative imaging | Automatic pediatric bone age and developmental assessment; delivers precise adult height and bone age estimation based on the Greulich and Pyle atlas |
| [AZmeasure](https://www.azmed.co/) | AZmed SAS | AI | Radiology | Quantitative imaging | CE mark-approved software solution; provides automated characterization of osteo-articular geometries, incl. lengths and angular positions; supports scoliosis, hallux valgus, flat / hollow foot, leg length discrepancies, hip dysplasia, and FAI |
| [BoneMetrics](https://www.gleamer.ai/solutions/bonemetrics) | Gleamer | AI | Radiology | Quantitative imaging | CE mark-approved software, which automates MSK measurements on x-ray, streamlining radiological workflows; fully automates standard measurements for feet, legs, pelvis / hip, and spine |
| [BoneAge](https://www.gleamer.ai/solutions/boneage) | Gleamer | AI | Radiology | Quantitative imaging | CE mark-approved software, automating bone age assessment using the Greulich & Pyle atlas method |
| [CoLumbo](https://columbo.me/) | Smart Soft Healthcare | AI | Radiology | Quantitative imaging | FDA-approved post-processing and measurement software; provides quantitative spine measurements from lumbar spine MRIs, incl. feature segmentation, threshold-based labeling of out-of-range measurements, and export of written report; tool does not produce a diagnosis/treatment recommendation |
| [AI-Rad Companion MSK](https://www.siemens-healthineers.com/de-ch/digital-health-solutions/ai-rad-companion) | Siemens Healthineers | AI | Radiology | Quantitative imaging | Image processing software providing quantitative and qualitative analysis from CT images to support physicians in the evaluation and assessment of MSK diseases; automatically detects and quantifies MSK-related abnormalities; provides segmentation / labeling of vertebrae, measurements of height in each vertebra and indication if they are critically different, and measurement of Hounsfield values in volumes of interest within vertebrae |
| [SMART Bun-Yo-Matic](https://paragon28.com/products/smart-bun-yo-matic/) | Disior Ltd. | AI | Radiology | Treatment planning | FDA-cleared software for diagnosis and surgical planning based on x-rays or weight-bearing CTs; generates 3D patient-specific case report; allows for 3D preoperative planning and analysis from 2D x-ray imaging; identifies recommended correction for hallux valgus and detects presence of abnormal anatomy; designed to be used with ‘Bun-Yo-Matic Lapidus Clamp’ surgical instrument |
| [HealthJOINT](https://www.accessdata.fda.gov/cdrh_docs/pdf20/K202487.pdf) | Zebra Medical Vision Ltd. | AI | Radiology | Treatment planning | Software for 3D reconstruction of bone from a set of 2D radiographs; assists in preoperative planning of knee orthopedic procedures; provides list of anatomical landmarks with their position on the 3D model |
| [Precision AI surgical planning system](https://www.precisionai.com.au/products/) | Precision AI Pty Ltd. | AI | Radiology | Treatment planning | FDA-cleared, AI-enabled patient-specific surgical planning for shoulder arthroplasty; software generates a surgery report with presurgical plan data file; hardware (guides and biomodels) intended as patient-specific surgical instruments to assist with intraoperative positioning |
| [PreView Shoulder](https://www.accessdata.fda.gov/cdrh_docs/pdf21/K210556.pdf) | Genesis Software Innovations | AI | Radiology | Treatment planning | FDA-cleared tool to develop preoperative shoulder plans based on CT scans; generates a report; solution allows the user to digitally perform the surgical planning by showing a representation of the patient’s shoulder anatomy as a 3D model and allows the surgeon to place the implant |
| [Virtual Implant Positioning System (VIP)](https://www.arthrex.com/arthroplasty-shoulder/virtual-implant-positioning-vip-system) | Arthrex Inc. | AI | Radiology | Treatment planning | Advanced preoperative planning tool for shoulder arthroplasty; uses CT scans of patient’s anatomy to create detailed 3D models for preoperative planning; key features include implant selection and positioning simulation, ROM analysis, and osteophyte removal planning; system received FDA clearance |
| [Blueprint](https://www.stryker.com/us/en/trauma-and-extremities/products/blueprint.html) | Stryker Corp. | AI | Radiology | Treatment planning | Advanced, surgeon-controlled tool for treatment planning in shoulder arthroplasty, combining 3D preoperative planning software with mixed reality guidance; provides optional patient-specific instrumentation; enables accurate glenoid positioning, improves decision-making by enhancing surgeon’s understanding of deformities, and obtains precise and reproducible 3D measurements; latest iteration combines software with Microsoft’s HoloLens 2 headset to provide intra-operative mixed reality guidance |
| [Ortoma Treatment Solution (OTS)](https://ortoma.com/) | Ortoma AB | AI | Radiology | Treatment planning | FDA- and CE mark-approved integrated AI platform designed to provide surgical planning, surgical navigation, post-operative verification, and follow-up; according to company’s website, the system drastically reduces the time needed for pre-operative planning in 3D; provides an automated AI analysis, incl. bone segmentation, landmarks, implant size, and implant position, which serves as the basis for pre-operative planning |
| [United orthopedic knee patient specific instrumentation](https://www.enhatch.com/intelligent-surgery-knee) | Enhatch Inc. | AI | Radiology | Treatment planning | FDA-cleared software solution; creates patient-specific instrumentation using either x-ray or CT images; streamlines surgical planning |
| [Acorn 3D Software](https://www.accessdata.fda.gov/cdrh_docs/pdf23/K234009.pdf) | Mighty Oak Medical | AI | Radiology | Treatment planning | Software tool for automatic segmentation, measuring, and treatment planning; output can be used to fabricate physical replicas using additive manufacturing; physical replicas can be used for diagnostic purposes in MSK care / craniomaxillofacial applications |
| [Axial3D Insight](https://axial3d.com/insight-platform/) | Axial Medical Printing Ltd. | AI | Radiology | Other | Cloud-based service and image segmentation framework; output file can be used for the fabrication of physical replicas using additive manufacturing (can be used for treatment planning/diagnostic purposes) |
| [SubtleSYNTH](https://subtlemedical.com/subtlesynth/) | Subtle Medical Inc. | AI | Radiology | Other | Software for synthesizing “SynthSTIR” contrast images from T1- and T2-weighted spine MRIs |
| [aprevo Digital Segmentation](https://carlsmed.com/aprevo-technology-platform/) | Carlsmed Inc. | AI | Radiology | Other | Software to perform digital image segmentation of the spine; device inputs DICOM images and outputs a 3D model of the spine |
| [HipCheck](https://www.accessdata.fda.gov/cdrh_docs/pdf23/K230045.pdf) | Stryker Corp. | AI | Radiology | Other | Assists in determining quantitative measurements for FAI-related procedures; provides static localization information derived from image processing of intraoperatively acquired static fluoroscopic images, by superposition of virtual measurement tools onto those x-ray images; ‘HipMap FAI Analysis’ is a patient-specific report used to support preclinical decision making; provides a morphological analysis of hips with potential FAI, including measurements and visualizations that describe hip impingement and stability |
| [Spine CAMP](https://www.accessdata.fda.gov/cdrh_docs/pdf22/K221632.pdf) | Medical Metrics Inc. | AI | Radiology | Other | Fully automated software; analyzes x-ray images of the spine to produce reports containing static and/or motion metrics; can be used to visualize intervertebral motion; metrics can be used to assess spinal health |
| [ENDEX, ENCOG](https://enlitic.com/) | Enlitic Inc. | AI | Radiology | Other | Enlitic ENDEX transforms medical imaging data to a consistent, clinically relevant standard nomenclature, enabling consistent display of hanging protocols and improving image routing; ENCOG uses AI to anonymize protected health information while maintaining clinically relevant data |
| [ARVIS](https://enovis.com/arvis) | Enovis Corp. | AI | Radiology | Other | FDA-cleared augmented reality system, comprising a wearable device and accompanying software, delivering hands-free, real-time surgical guidance; cameras mounted on the device track markers on the patient to provide accurate navigation guidance for total hip, total knee, and unicompartmental knee arthroplasty |
| [myExam Companion](https://www.siemens-healthineers.com/medical-imaging/digital-transformation-of-radiology/myexam-companion) | Siemens Healthineers | AI | Radiology | Other | Uses AI to standardize patient positioning and scan planning in MRI / CT, reducing operator-dependent errors and scan times; helps users achieve reproducible results; guides operators through diagnostic procedures |
| [Orthelligent Vision](https://en.o-dhs.com/produkt/orthelligent-vision/) | OPED GmbH | AI | Imaging | Gait analysis | FDA- and CE-registered smartphone / tablet app for precise, AI-based, marker-less gait analysis; measurements can be acquired in < 5 minutes; automatic, detailed report generation with intuitive visualizations; supports frontal and lateral analyses, as well as analysis of stair descent; targeted at physiotherapists, doctors, and orthopedic technicians |
| [Momentum Spine](https://momentum.health/) | Momentum Health Inc. | AI | Imaging | Contour sensing | Optical contour sensing mobile application intended to quantify asymmetries, assess body angles, and curve progression related to postural asymmetries, incl. scoliosis; available ‘over the counter’, but may also be prescribed; enables remote management of scoliosis; scans take < 30 seconds; enables tracking of parameters over time |
| [RadioReport Automatic AI](https://radioreport.com/musculoskeletal-radiology/) | Neo Q Quality in Imaging GmbH | AI | Radiology | Clinical documentation | Uses 23 anatomical modules (incl. MSK MRI) instead of pathology-specific templates; achieves 50% faster reporting via guided virtual interviews and mandatory plausibility checks; generates machine-readable reports compatible with big data analytics; company claims from image to report in < 80 seconds |
| [Rad AI Suite](https://www.radai.com/reporting) | Rad AI | AI | Radiology | Clinical documentation | AI-driven dictation solution; according to company website, reduces dictation words by 90% through adaptive generative AI that learns individual radiologists’ styles; automates summary generation with 95% unedited acceptance rate in production environments; flags actionable findings through automated follow-up management |
| *Therapeutic software / devices and rehabilitation* | | | | | |
| [Companion Patella](https://www.medi.de/produkte/diga-companion-patella/) | Medi GmbH & Co. KG | AI/DH | Therapeutic / rehabilitation | n/a | Digital health application designed for patients with specific knee pain around the patella; key features include an individualized therapy program, AI-based algorithm, training statistics, and professional knowledge base; web/smartphone app; indicated for specific knee conditions, including patellar tendinopathy (‘jumper’s knee’), patellofemoral pain syndrome, and (first-time) patellar dislocation; approved as a ‘DiGA’ in Germany, classified as a Class I medical device under the EU’s MDR; validated in collaboration with specialists from the patellofemoral committee of the German Knee Society; recommended usage is approx. 25 minutes per therapy session, with daily training provided; app is available in German and free for patients with a prescription, costing insurance providers €223.49 for a 90-day treatment period |
| [Companion Shoulder](https://www.medi.de/produkte/diga-companion-shoulder/) | Medi GmbH & Co. KG | DH | Therapeutic / rehabilitation | n/a | Digital health application designed for patients with shoulder complaints; provides therapeutic training program for individuals suffering from shoulder lesions, discomfort, or pain; key features include personalized therapy, video-guided exercises, adaptive training, and educational content in the form of medical knowledge articles; prescribed by a physician after diagnosis (‘M75 Shoulder lesions’); recommended usage is 3-7 times per week, with each session lasting 15-30 minutes; total duration of use is typically 90 days, unless otherwise recommended; approved as a ‘DiGA’ in Germany, classified as a Class I medical device under the EU’s MDR; cost of €419 per patient |
| [eCovery](https://ecovery.de/) | eCovery GmbH | DH | Therapeutic / rehabilitation | n/a | Digital health application designed to help patients with lower back pain; provides a personalized 12-week training program with video exercises that can be done at home; app adapts to each user’s health condition, tracks progress, and maintains a pain diary; key features include personalized training plans, initial assessment considering individual pain levels and physical limitations, and continuous adjustment of the training plan based on user feedback on pain values and exercise difficulty; classified as a Class I medical device under the EU’s MDR, received approval as a ‘DiGA’ in Germany; cost of €574 |
| [Mawendo](https://www.mawendo.com/) | Mawendo GmbH | DH | Therapeutic / rehabilitation | n/a | Digital health application that provides personalized therapy programs for patients with knee pain, specifically targeting patellofemoral pain syndrome; offers an exercise program with instructional videos, health information, and documentation options; provides a 12-week therapy program that allows patients to train independently, regardless of location and time; received approval as a ‘DiGA’ in Germany; cost of €119 |
| [Orthopy](https://www.orthopy.de/) | Orthopy Health GmbH | DH | Therapeutic / rehabilitation | n/a | Digital health application designed to support patients with knee injuries, specifically ACL tears and meniscus damage; provides guided physiotherapeutic training and educational content to help patients advance their recovery independently; offers personalized therapy plans, exercise videos, and progress tracking; accompanies patients before, during, and after orthopedic treatment; available in German; approved as a ‘DiGA’ in Germany, classified as a Class I medical device under the EU’s MDR; cost of €487.84 |
| [Vivira](https://www.vivira.com/) | Vivira Health Lab GmbH | AI/DH | Therapeutic / rehabilitation | n/a | Digital health application designed to provide targeted training sessions for reducing back pain; offers AI-powered physiotherapy programs that adapt dynamically based on user feedback; provides 15-minute daily exercise sessions, four times a week; uses video demonstrations and user feedback surveys to adjust exercise type and intensity; allows users to track their progress through activity history, charts on pain, mobility, and fitness; approved as a ‘DiGA’ in Germany, classified as a Class I medical device under the EU’s MDR; cost of €206.79 |
| [Thrive](https://swordhealth.com/solutions/thrive) | Sword Health Inc. | AI/DH | Therapeutic / rehabilitation | n/a | Digital physical therapy application designed to help users manage MSK conditions from home; key features include personalized treatment, technology-assisted therapy, and clinical support; uses AI to provide real-time feedback during exercise sessions; recently introduced an AI assistant called ‘Phoenix’, which uses natural conversation to guide and react to patients during their physical therapy sessions; AI analyzes patient progress and performance data, identifying trends and generating actionable insights for human clinicians |
| [Hinge Health](https://www.hingehealth.com/) | Hinge Health Inc. | AI/DH | Therapeutic / rehabilitation | n/a | Comprehensive digital MSK care solution; platform combines technology, AI, and clinical expertise to provide personalized care for individuals with back, joint, and muscle pain; core offering is a ‘digital MSK clinic’ that provides personalized exercise therapy, education, and support; uses computer vision for advanced motion-tracking capabilities; offers real-time exercise guidance and feedback; platform uses AI to create dynamically personalized care plans |

*Abbreviations: AI, artificial intelligence; DH, digital health; ML, machine learning; DL, deep learning; MSK, musculoskeletal; ROM, range of motion; MDR, medical device regulation; FDA, U.S. Food and Drug Administration; HIPAA, Health Insurance Portability and Accountability Act; GDPR, General Data Protection Regulation; EHR, electronic health record; CT, computed tomography; ACL, anterior cruciate ligament; MRI, magnetic resonance imaging; DiGA, Digitale Gesundheitsanwendung*.
